# A Sequent of Gram-Negative Co-Infectome-Induced Acute Respiratory Distress Syndrome Are Potentially Subtle Aggravators Associated to the SARS-CoV-2 Evolution of Virulence

**DOI:** 10.3390/diagnostics14010120

**Published:** 2024-01-04

**Authors:** Kamaleldin B. Said, Ahmed Alsolami, Khalid F. Alshammari, Fawaz Alshammari, Sulaf A. Alhallabi, Shahad F. Alafnan, Safia Moussa, Abdelhafiz I. Bashir, Kareemah S. Alshurtan, Rana Aboras, Ehab K. Sogeir, Alfatih M. A. Alnajib, Abdullah D. Alotaibi, Ruba M. Elsaid Ahmed

**Affiliations:** 1 Department of Pathology, College of Medicine, University of Ha’il, Ha’il 55476, Saudi Arabiarm.ahmed@liveuohedu.onmicrosoft.com (R.M.E.A.); 2 Genomics, Bioinformatics and Systems Biology, Carleton University, 1125 Colonel-By Drive, Ottawa, ON K1S 5B6, Canada; 3 Department of Internal Medicine, College of Medicine, University of Ha’il, Ha’il 55476, Saudi Arabia; 4 Department of Dermatology, College of Medicine, University of Ha’il, Ha’il 55476, Saudi Arabia; 5 Department of Microbiology, King Salman Specialist Hospital, Ha’il 55476, Saudi Arabia; safiamoussa89@yahoo.com; 6 Department of Physiology, College of Medicine, University of Hail, Ha’il 55476, Saudi Arabia; 7 Departments of Intensive Care, College of Medicine, University of Ha’il, Ha’il 55476, Saudi Arabia; 8 Department of Family and Community Medicine, College of Medicine, University of Ha’il, Ha’il 55476, Saudi Arabia; 9 Department of Surgery, College of Medicine, University of Ha’il, Ha’il 55476, Saudi Arabia; 10 Department of Otolaryngology, College of Medicine, University of Ha’il, Ha’il 55476, Saudi Arabia; adqo@yahoo.com

**Keywords:** ARDS, co-infections, COVID-19 fatality, co-evolution of virulence

## Abstract

Acute respiratory distress syndrome (ARDS) is one of the major problems in COVID-19 that is not well understood. ARDS is usually complicated by co-infections in hospitals. Although ARDS is inherited by Europeans and Africans, this is not clear for those from the Middle East. There are severe limitations in correlations made between COVID-19, ARDS, co-infectome, and patient demographics. We investigated 298 patients for associations of ARDS, coinfections, and patient demographics on COVID-19 patients’ outcomes. Of the 149 patients examined for ARDS during COVID-19, 16 had an incidence with a higher case fatality rate (CFR) of 75.0% compared to those without ARDS (27.0%) (*p* value = 0.0001). The co-infectome association showed a CFR of 31.3% in co-infected patients; meanwhile, only 4.8% of those without co-infections (*p* value = 0.01) died. The major bacteria were *Acinetobacter baumannii* and *Escherichia coli*, either alone or in a mixed infection with *Klebsiella pneumoniae*. Kaplan–Meier survival analysis of COVID-19 patients with and without ARDS revealed a significant difference in the survival time of patients with ARDS (58.8 +/− 2.7 days) and without ARDS (41.9 +/− 1.8 days) (*p* value = 0.0002). These findings prove that increased hospital time was risky for co-infectome-induced SDRS later on. This also explained that while empiric therapy and lethal ventilations delayed the mortality in 75% of patients, they potentially did not help those without co-infection or ARDS who stayed for shorter times. In addition, the age of patients (*n* = 298) was significantly associated with ARDS (72.9 +/− 8.9) compared to those without it (56.2 +/− 15.1) and was irrespective of gender. However, there were no significant differences neither in the age of admitted patients before COVID-19 (58.5 +/− 15.3) and during COVID-19 (57.2 +/− 15.5) nor in the gender and COVID-19 fatality (*p* value 0.546). Thus, Gram-negative co-infectome potentially induced fatal ARDS, aggravating the COVID-19 outcome. These findings are important for the specific differential diagnosis of patients with and without ARDS and co-infections. Future vertical investigations on mechanisms of Gram-negative-induced ARDS are imperative since hypervirulent strains are rapidly circulating. This study was limited as it was a single-center study confined to Ha’il hospitals; a large-scale investigation in major national hospitals would gain more insights.

## 1. Introduction

Acute respiratory distress syndrome (ARDS) has been regarded as a major problem in the diagnosis of lung diseases [1]. The features that can define ARDS include poor oxygenation, pulmonary infiltrates, and early onset scenarios featuring the hallmarks of the ARDS (this form will be used hereafter), wherein the PaO_2_/FiO_2_ ratio drops to under 300. The Berlin definition of ARDS [2] was modified in 2012, where the term “acute lung injury” was excluded. A draft definition proposed three mutually exclusive categories of ARDS based on degree of hypoxemia: mild (200 mm Hg < PaO_2_/FIO_2_ ≤ 300 mm Hg), modrate (100 mm Hg < PaO_2_/FIO_2_ ≤ 200 mm Hg), and severe (PaO_2_/FIO_2_ ≤ 100 mm Hg), as well as four ancillary variables for severe ARDS: radiographic severity, respiratory system compliance (≤40 mL/cm H_2_O), positive end-expiratory pressure (≥10 cm H_2_O), and corrected expired volume per minute (≥10 L/min) [3]. Consequently, few effective therapy approaches exist to treat ARDS, which has a significant fatality rate [3,4]. Some studies have raised doubts about the notion that diverse events result in identical scenarios [5,6,7]. Therefore, the mechanism(s) and pre-disposing factors involved in ARDS, and, particularly, how it is stimulated during COVID-19 are not clear. Thus, it has become imperative to understand the rates and frequencies of ARDS before and after the COVID-19 pandemic.

The epidemiology of ARDS is important in understanding the different mechanisms of its evolution. ARDS was first identified by Ashbaugh et al. [1] in 1967; however, it still remains a significant risk of mortality globally [8,9,10]. The recent Berlin definition [2] is more improved, albeit variability exists in different settings [8]. In fact, the incidence of ARDS ranges from 1.5 cases per 100,000 to nearly 79 cases per 100,000 [9]. with European countries reporting a lower incidence than the USA [10] Moreover, studies from Brazil reported incidence rates ranging from 1.8 to 31 per 100,000 [11,12].

Although the overall survival rate is increasing [13,14], the in-hospital mortality rate varies significantly across a number of observational studies [8,9,13,15,16,17]. This might be accounted for by variations in risk factors, diagnostic accessibility, awareness of ARDS, and some selection biases impacting clinical trials [18]. The incidence of ARDS was recently assessed across 459 intensive care units (ICUs) in 50 countries as part of a major worldwide observational research (the LUNG SAFE trial) [19]. In the aforementioned report, ARDS occurred in 10.4% of all ICU admissions and in 23.4% of patients requiring mechanical ventilation among 4499 patients who developed acute hypoxemic respiratory failure. In comparison to South America, Asia, and Africa, higher incidence rates were found in North America, Oceania, and Europe. According to the Berlin criteria, 30.0% of patients had mild ARDS, 46.6% had moderate, and the remaining 23.4% had severe ARDS. The LUNG SAFE trial’s adjunctive therapies and ventilator management were among its secondary endpoints, the use of optimal mechanical ventilation was low, and even adjunctive treatments were underutilized for ARDS patients [20,21,22]. Thus, the severity of ARDS worsened in 19% of patients, in-hospital mortality was 40%, and fatality increased concurrently with increasing pressure, reaching 46% for severe ARDS [19].

There are over 60 probable predisposing risk factors for SARS-CoV-2-induced ARDS; the most frequent were attributed to a small number of prevalent causes including septic pneumonia [23,24] The widely studied etiologies are pneumonia (40%), sepsis (32%), and aspiration (9%), as reported on 107 patients in a medical intensive care unit [25]. Some known risk factors are prone to stimulate ARDS indirectly. These include pneumonia as the most common cause outside the hospital [26] in the form of community-acquired pneumonia with alarming rates of increasing mortality of up to 25%. *Streptococcus pneumoniae* [27], *Legionella pneumophila*, *Pneumocystis jirovecii*, *Staphylococcus aureus*, enteric gram-negative organisms, and several respiratory viruses are examples of common pathogens [28,29]. Additionally, nosocomial pneumonia can develop into ARDS, most frequently caused by *Staphylococcus aureus*, *Pseudomonas aeruginosa*, and other enteric gram-negative bacteria. Community-acquired MRSA (CA-MRSA) pneumonia is thought to be the major etiology of necrotizing pneumonia-induced ARDS in the past decade. Intensive care unit admission and in-patient mortality were much higher for patients with CA-MRSA pneumonia than for those with pneumococcal CAP [30]. According to some studies, the fatality rate for CA-MRSA pneumonia might range from 56% to 63% [31,32]. The pathogenicity of Panton–Valentine leukocidin (PVL) is frequently linked to CA-MRSA strains [33]. Extensive lung necrosis, multi-lobular infiltrates, leucopenia, hemoptysis, and sepsis are symptoms of CAP caused by MRSA bearing the PVL gene, which increases the mortality rate [34,35]. 

The second most common trigger of ARDS is sepsis [36,37]. Community-acquired pneumonia (CAP) is most severe in communities and/or extended home care worldwide due to the septic necrotizing infection [38,39]. *Staphylococcus aureus* has been known to induce ARDS for years. The MRSA’s direct involvement through FTY720 S-phosphonate in endothelial cell protection was confirmed [40]. There are about 30 million cases of lung sepsis per year and over eight million deaths, i.e., 15–30% in high-income countries and 50% or higher in low- and middle-income countries [41]. It becomes critically serious when *pvl*-positive CA-MRSA lineages are involved in ARDSARDS. However, data are limited on these new aspects of COVID-19-complicated bacterial infections. Despite the tremendous advances in healthcare systems, respiratory problems still remain a major issue [42,43]. Particularly, lung-related problems incur significant costs and are foreseen to increase with increasing microbial resistance and the world’s aging population [44]. In the US alone, the annual hospitalization rate for CAP was more than USD 2.6 million, ranking second only to childbirth for hospital admissions (available at: https://www.hcup-us.ahrq.gov/db/nation/nis/NIS_Introduction_2017.jsp, accessed on 24 May 2022, the Agency for Healthcare Research and Quality (and quality) National: regional estimates on hospital use for all patients from the HCUP National Inpatient Survey (NIS 2017)). Therefore, a leading cause of death worldwide is sepsis, especially when developed as a dysregulated immune response to infectious pneumonia [45,46]. The potential risk of *S. aureus* in these cases is quite high.

COVID-19 was one of the main reasons for ARDS during the pandemic that worsened outcomes. Early studies conducted to characterize the COVID-19 host immune response showed an immunological signature comprised of many serum cytokines [47,48]. Compared to other viruses, COVID-19 and influenza are both linked to a compromised IFN-I and -III host response. However, the severity of the impairment is inversely correlated with COVID-19. In addition, age has emerged as a dominating predictor of illness severity and mortality risk, even though much of the precise mechanisms remain unknown. Early on in the epidemic, reports from China and Italy indicated case-fatality rates of 15–20% among patients over the age of 80 compared to 1% among patients under the age of 50 [49] and concluded that COVID-19 ARDS appeared to have a worse outcome than ARDS from other causes. Patients with COVID-19 ARDS who were hospitalized in the ICU experienced mortality rates ranging from 26% to 61.5%, and patients receiving mechanical ventilation experienced mortality rates ranging from 65.7% to 94%. However, numerous studies have demonstrated that the pathophysiological characteristics of COVID-19 ARDS are equal to those of non-COVID-19 ARDS [50]. These findings indicate that there is a knowledge gap in these areas of research.

Thus, specific diagnosis of COVID-19-mediated, bacterial infectome-induced, or non-infectious ARDS syndromes has become imperative for the clinical management of patients. These overlapping mechanisms have required clinicians to ponder over several of the scenarios involved, particularly, in cases requiring immediate interventions without laboratory aid. The diagnosis of ARDS cannot be confirmed or disproven by a single diagnostic test, which adds another layer of difficulty. Furthermore, according to the Berlin definition’s expansion, it must be emphasized that ARDS is currently diagnosed only based on clinical criteria and is a syndrome rather than a particular pathologic entity until one is specifically identified. Therefore, the diagnosis of ARDS necessitates the presence of bilateral chest radiographic abnormalities and new or worsening respiratory distress for seven days or less, as well as the inability of heart failure to fully explain the hypoxemia and the radiographic infiltration and clinical significance of the impaired oxygenation. In contrast to earlier definitions [51], the Berlin criteria offered more precise guidance on chest radiograph patterns that are indicative of ARDS. There is a consensus that the presence of ground glass opacities (GGOs) is the key CT characteristic of COVID-19 pneumonia often observed with an absence of centrilobular nodules and mucoid impactions that makes the characteristics of COVID-19 pneumonia unique [52,53]. Nevertheless, there is currently a lack of pathological data on COVID-19 pneumonia based on autopsy or biopsy results. Furthermore, for patients with severe hypoxia and those on high doses of vasoactive drugs, continuous renal replacement therapy, or other ICU procedures, obtaining a CT scan can be difficult. Moreover, CT is costly, and exposure to ionizing radiation limits its repeatability [54,55]. In the Kigali update to the Berlin definition of ARDS, lung ultrasonography has been suggested as a substitute for chest radiography in settings with limited resources. Combining cardiac and lung ultrasonography can suggest a cardiogenic process, although heart failure and ARDS can coexist complicating the issue. Ultrasound visualizes primarily subpleural lung zones and can yield poor-quality images in the presence of extensive overlying soft tissue (as seen with obesity) or subcutaneous oedema [56,57]. Thus, for these and other several reasons, baseline association studies, such as this work, are required to lay solid foundations for the rapid primary differential diagnosis of respiratory illnesses, with an emphasis on COVID-19-induced and bacterial co-infectome-induced distresses. This study aimed to conduct a comprehensive investigation for associations of ARDS, coinfections, and patient demographics on COVID-19 patients’ outcomes, with an emphasis on the potential influence of each specific diagnosis.

## 2. Materials and Methods

Hospital and laboratory records on different ARDS, COVID-19, and co-infection scenario data recorded pre-COVID-19 and during COVID-19 were collected. The nasal-pharyngeal swab test was taken as the pathognomonic test for the diagnosis of COVID-19. Since there is no clear-cut diagnostic procedure available for a one-step diagnosis of ARDS, multiple indicative criteria that are usually followed, including standard guidelines’ meeting definitions, were collected (tests shown below). SARS-CoV-2 was confirmed using swabs from nasopharyngeal secretions using a specific PCR test. In addition, a clinically compatible COVID-19 illness was confirmed through clinical history, epidemiological contact, and a qPCR test. COVID-19 ARDS is diagnosed after a confirmed PCR test for SARS-CoV-2 and compatibility with Berlin 2012 ARDS criteria, including (i) severe shortness of breath; (ii) onset of aggravating lung symptoms or known clinical insult in a week time; (iii) diffused bilateral confluent air space opacities (ground-glass) on chest X-ray, computed tomography (CT), or ultrasound which is not supported by effusions, lobar or lung collapse, or nodules; and (iv) if cardiac arrest is not the apparent reason for shortness of breath. These criteria identify unusual scenarios in the shortness of oxygen that are normally used to diagnose situations. Therefore, pre-COVID ARDS is familiar; however, its association with SARS-CoV-2 viral pneumonia and other microbial co-infections is not always successfully differentiated.

### 2.1. Study Designs

This study was a retrospective cross-sectional study using experimental records reported at the King Salman Specialist Hospital (KSSH), Ha’il, Kingdom of Saudi Arabia (KSA). We designed this investigation to understand the associations of ARDS, coinfections, and patient demographics on COVID-19 patients’ outcomes, with an emphasis on the potential influence of each on specific diagnoses. Although there is a long array of factors that influence ARDS, we focused on the above factors that are likely to aggravate, induce, or synergize SARS-CoV-2 fatality. All diagnostic criteria and reported test results as well as inclusion criteria were reviewed by a panel of experts. These COVID-19 patient records (*n* = 298) were used in the study to understand different aspects of ARDS and COVID-19 coinfections. This included associations of COVID-19 fatality rates among patients with and without ARDS, as well as a comparative analysis of multi-factors involved in patient outcomes and prognosis, including potential sources of induction of ARDS, the influence of coinfections with and without ARDS, and patient demographics. All these factors were examined before and after the COVID-19 pandemic to accurately understand the potential pathogenicity, mechanisms, and the likely source(s) of stimulants of ARDS in the COVID-19 context. To avoid confounding factors and experimental pitfalls, we applied rigorous data analysis. For instance, since infecting strains are usually clonal in nature, we used single isolates per patient, if they were isolated at the same time. For ICU patients, the average stay was around two to three weeks from admission. COVID-19 diagnosis for each participating patient was confirmed through a molecular diagnosis using real-time reverse transcription PCR (RT-PCR) testing performed on nasopharyngeal throat swab specimens at the Ha’il Health Regional Laboratory (HHRL) for COVID-19. Ethical approval for this project (number RG21074) has been reviewed and approved by the Research Ethical Committee (REC) of the University of Ha’il, dated 22 November 2021 under numbers H-2021-215, File H-2020-632-16160.

### 2.2. Supporting Examinations and Tests Performed for Confirmation

The features that can define ARDS include poor oxygenation, pulmonary infiltrates, and early onset scenarios, which are the hallmarks of the ARDS (this form will be used hereafter to mean both ARDS and ARDS), where the PaO_2_/FiO_2_ ratio drops to under 300. The Berlin definition of ARDS [2] was modified in 2012, where the term “acute lung injury” was excluded. A Berlin draft definition introduced three types of ARDS based on the level of deficient oxygenation, namely, mild (200 mm Hg < PaO_2_/FIO_2_ ≤ 300 mm Hg), moderate (100 mm Hg < PaO_2_/FIO_2_ ≤ 200 mm Hg), and severe (PaO_2_/FIO_2_ ≤ 100 mm Hg), as well as 4 ancillary variables for severe ARDS: radiographic severity, respiratory system compliance (≤40 mL/cm H_2_O), positive end-expiratory pressure (≥10 cm H_2_O), and corrected expired volume per minute (≥10 L/min) [2].

1. Oxygen: Non-invasive oxygenation was undertaken using supplemental oxygen in patients with signs of hypoxemia (i.e., SpO_2_ < 90%). Initially, 5 L/min was used, which was then titrated to SpO_2_ ≥ 90% as required. High oxygenation flowed (10–15 or 50–60 L/min) through a facemask that was attached to a restoration bag for an elevated oxygen level as reported by Borghes and Maroldi [54] Nava et al., 2011 [55] and Keenan et al., 2011 [56] the described procedures were initiated for reasonable levels then elevated gradually, namely, from nasal cannula (~4 L) to a simple facemask (~10 L), then a non-Rebreather mask (~15 L). As required, noninvasive ventilation was used for enhanced flow, e.g., a high-flow nasal cannula (100 L) or Bilevel-positive airway pressure (BiPAP).
2. Intubation: Mechanical ventilations increased difficulties with breathing or hypoxemia when needed. This was applied through an endotracheal tube or tracheostomy using an ICU expert according to the NIH NHLBI ARDS Clinical Network’s mechanical ventilation protocol card, available at: http://www.ardsnet.org/system/files/Ventilator%20Protocol%20Card.pd (accessed on 5 December 2021).
3. Lowest absolute lymphocyte count (LALC) and routine complete blood and differential counts performed by using laboratory-automated hematology analyzers according to Fan et al. (2020) [57] and Kaushansky et al., 2015 [58].
4. Records of microbial co-infection or superinfection and their antimicrobial susceptibility data during ARDS co-infections.

Routine microbiological investigation data from SDRS cases with clinical COVID-19 co-infecting pathogens were collected. Bacterial coinfectomes (co-infectomes) (bacterial pathogens co-infected with the SARS-CoV-2 virus) were studied on a case-by-case basis during the overall evaluations. The susceptibility testing results recorded in accordance with the recommendations of the Clinical and Laboratory Standard Institute (CLSI document M100S-26) [59,60] were used to categorize resistance classifications.

### 2.3. Statistical Analysis of the Data

Data from different sources and experimental procedures were analyzed using a statistical analysis program, namely, the Statistical Package for Social Sciences software (SPSS) (IBM SPSS; Version 24 SPSS version 23.0 for Windows (SPSS, Inc., Chicago, IL, USA). The analysis was descriptive in groups; Fisher and chi-square tests were used, and *p*-values were statistically significant if they were <0.05). Kaplan–Meier survival analysis was used to delineate the comprehensive survival outcomes of the complete study cohort, commencing from the moment of their hospital admission.

## 3. Results

**Figure 1:**
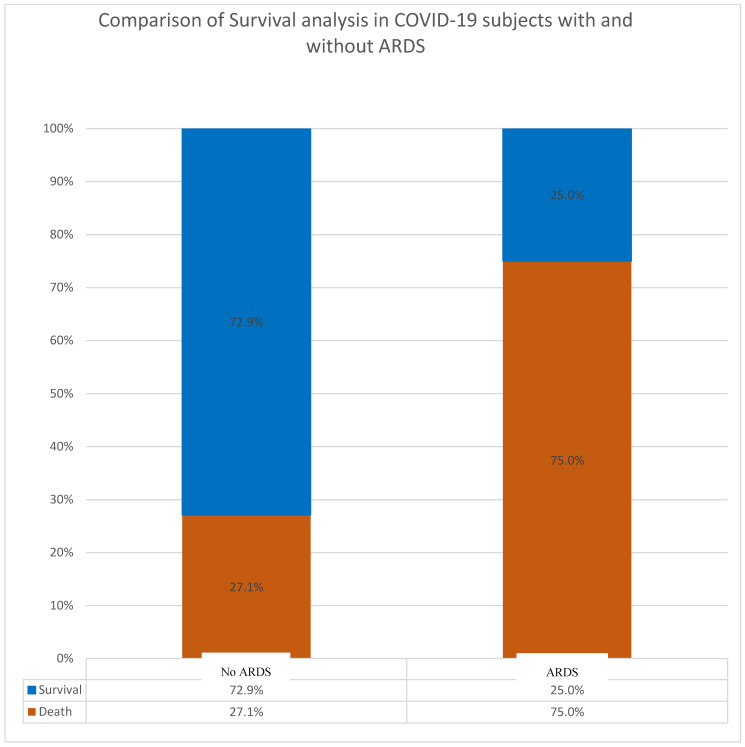
Comparison of survival analysis in COVID-19 subjects with no ARDS versus those with it.

We examined 298 COVID-19 patients for different types of factors that aggravate the disease with an emphasis on ARDS. In addition, we examined differences in patient demographics before and after the pandemic to understand gender-based susceptibility to COVID-19 and ARDS incidences. First, to understand the significance of the influences of ARDS and bacterial co-infections on COVID-19 fatality, we performed a survival analysis of COVID-19 patients with and without ARDS. Secondly, we carried out a comparison of survival analysis in COVID-19 patients with underlying ARDS who were either co-infected or not co-infected with bacterial pathogens. In the first analysis of the 149 patients examined for the presence or absence of ARDS, 16 had an ARDS disorder while 133 did not. The results indicated that death rates among COVID-19 patients with ARDS were much higher (75.0%) compared to COVID-19 patients without ARDS (27.0%). The association analysis of ARDS and COVID-19 fatality rates showed a highly significant value (*p* value = 0.000106022666010979). This indicated that 73% of patients without the disorder ARDS survived COVID-19 infection (Figure 1, Table 1f).

**Figure 2:**
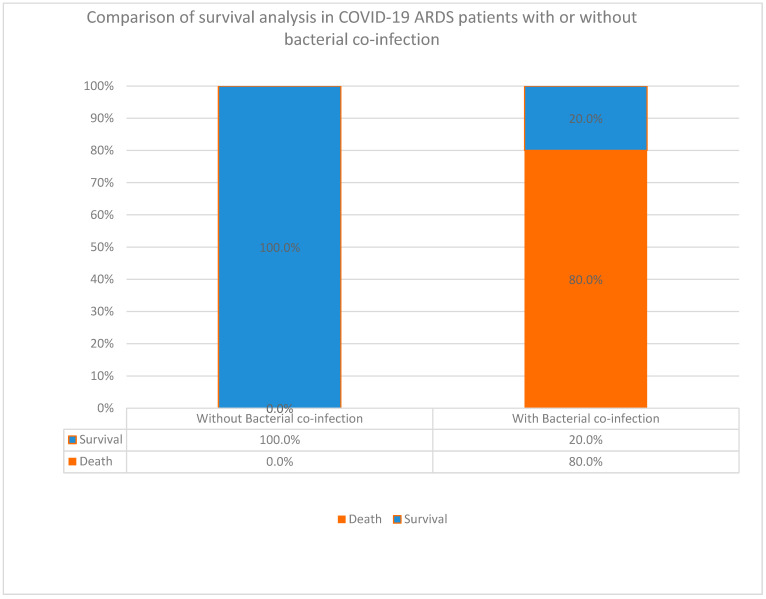
Comparison of survival analysis in COVID-19–ARDS patients with or without bacterial co-infection.

One of the major observations was the aggravation of COVID-19 with elevated fatality rates in cases of SARS-CoV-2 with bacterial co-infections. To provide further proof of concept, we studied the influence of bacterial co-infections in these patients. The results demonstrated that in co-infected COVID patients with underlying ARDS, significantly higher fatality rates were found. However, the association between bacterial co-infection and death rate was not statistically significant (*p* value = 0.250) (Figure 2). This was mainly due to the fact that only one patient had a combination of COVID and ARDS without bacterial co-infection. Therefore, the sample size was too small for comparison and further indicated bacterial co-infections in fatality rates aggravating the disease. The major bacterial pathogens that potentially predisposed one to ARDS were *Acinetobacter baumannii*, and *Escherichia coli* (*E. coli*), which, either alone or in a mixed infection with *Klebsiella pneumoniae* (*K. pneumoniae*), were predominant species identified during ARDS attacks. Typical patient characteristics and COVID-19 diagnostic features were evident in the influence of these pathogens in the aggravation of ARDS–COVID-19. The major exacerbating pathogens were *K. pneumoniae* and *A. baumannii*; both had much higher levels of lethal oxygenation (intubation) much more than 4 L, much lower levels of LALC than 5, and always produced lung infiltrations with ground glass on X-ray images (Table 1g–j).

**Figure 3:**
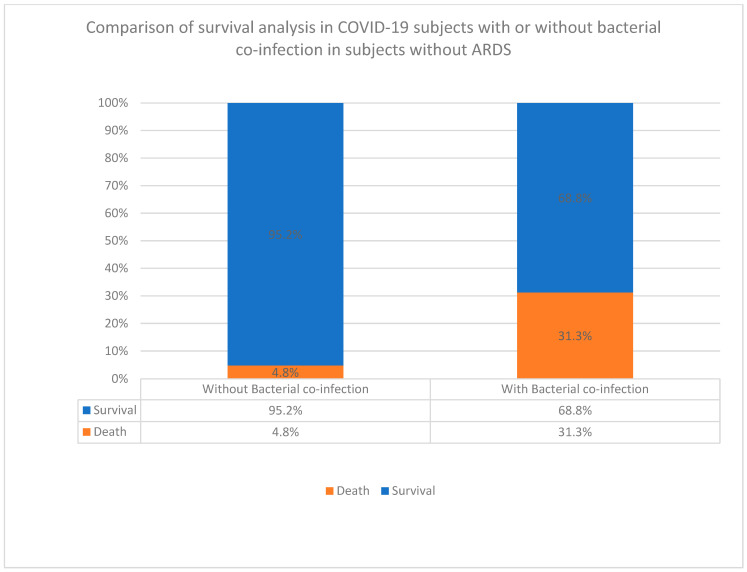
Comparison of survival analysis in COVID-19 subjects with or without bacterial co-infection in subjects without ARDS.

To provide further proof of concept in the significance of bacterial co-infections in COVID-19 patients without ARDS, we carried out an overall survival analysis in patients who had no ARDS (*n* = 133) conditions. In these patients with COVID and co-infection, 31.3% died, while only 4.8% died in those patients without bacterial co-infection. In other words, 95% of patients without co-infections survived the COVID-19 disease. This association between co-infection and COVID-19 fatality was highly associated with an increased death rate as indicated by a *p* value of 0.0121753241070998) (Figure 3).

**Figure 4:**
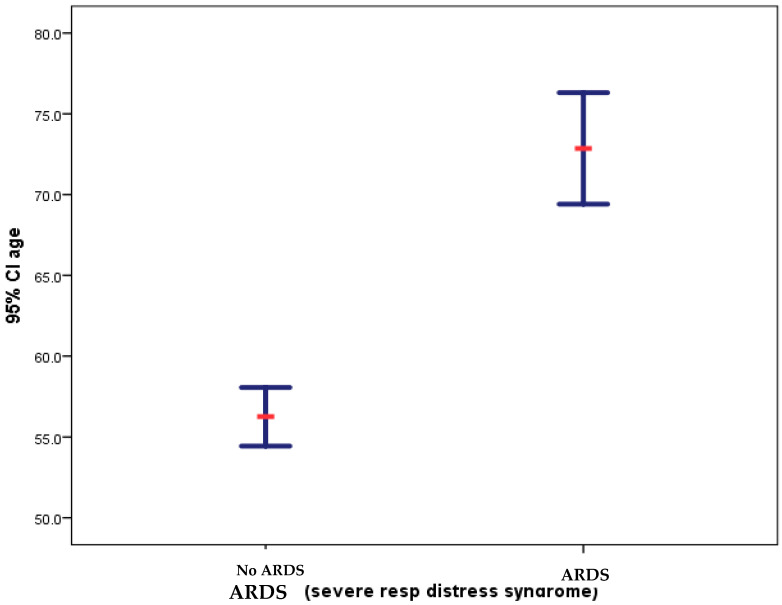
Association of admitted patients’ age with ARDS incidence rates in the study population in Ha’il, Saudi Arabia.

**Figure 5:**
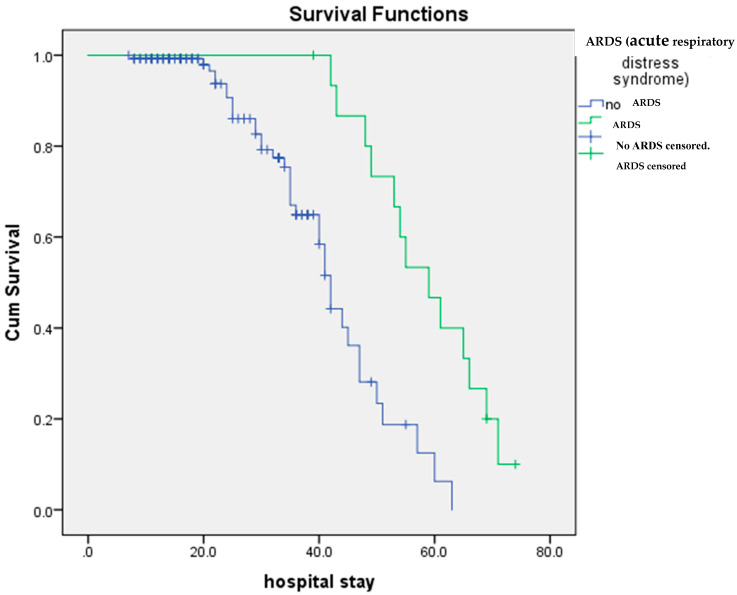
Kaplan–Meier survival analysis of COVID-19 patients with and without ARDS.

To test the notion that a specific age range or age group could be a potential risk factor in susceptibility to ARDS attack, we examined the ages of the target population (149 patients) (see Figure 2 for details). There was a significant difference in the age of patients with ARDS (72.9 +/− 8.9) compared to patients without ARDS (56.2 +/− 15.1) (Figure 4). More importantly, since hospital stay-time is a significant risk factor for ARDS development, to understand the hospital-stay time span until outcome on survival rates of patients (hospital stay in days), we carried out a Kaplan–Meier survival analysis on a total of 148 COVID-19 patients with and without ARDS (Figure 5). The results of this analysis revealed a significant difference in the survival time of patients with COVID-19 and ARDS (58.8 +/− 2.7 days) compared with those with COVID-19 and without ARDS (41.9 +/− 1.8 days) (*p* value = 0.000209700314444779). Patients with ARDS stayed for a prolonged period (delayed mortality) (~63 days) than those without the disorder (~42 days).

To confirm whether these findings could suggest an age-specific susceptibility factor selected by SARS-CoV-2, we analyzed the age factor before and during COVID-19 in all admitted patients (*n* = 298); there was no significant difference in the age of admitted patients before COVID-19 (58.5 +/− 15.3) and during COVID-19 (57.2 +/− 15.5) (Table 1a). Similarly, we further examined the probability of the influence of gender differences in COVID-19 patients with underlying ARDS (Table 1b). However, to avoid bias in result interpretations, we first examined the notion of female gender-based resistance to SARS-CoV-2. Among the study population of 298 patients, we did not find any significant association between the studied patients’ gender and COVID-19 disease incidence (Table 1b; *p* value 0.546). To avoid potential confounders, we also studied the relationships between the gender of the target population and ARDS cases (Table 1c); no significant association was found (*p* value 0.307). To rule out any association between case fatality and gender, we analyzed associations between the gender of patients and death and found no significant association (*p* value 0.433) (Table 1d). Furthermore, the probability that ADRS predisposes one to coinfection was also remote (Table 1e); (Pearson chi-square, *p* value 0.077) was insignificant. However, when we reversed dependents, there was a highly significant association with deaths, as explained above.

## 4. Discussion

In this study, we examined different factors that potentially aggravated COVID-19 to ultimately understand the mechanisms of the pathogenicity and virulence of the virus under different underlying conditions. A total of 298 COVID-19 patients were studied for different types of factors that exacerbated the disease with an emphasis on ARDS and patient demographics before and during the pandemic and to understand gender-based susceptibility to COVID-19 and ARDS incidences. First, to determine the significance of ARDS and bacterial co-infections on COVID-19 fatality, we performed a survival analysis of COVID-19 patients with and without ARDS. Secondly, we carried out a comparison of survival analysis in COVID-19 patients with underlying ARDS who were in two groups: co-infected and not co-infected with bacterial pathogens.

The elevated death rates obtained in this study (75%) among COVID-19 patients with ARDS compared to only 27% fatality rates on those without ARDS indicated that the disorder was a highly significant aggravating factor in the virus virulence with a highly significant value (*p* value = 0.000106022666010979). The death rate was much higher than that reported in other countries [61]; however, those reported in our study were carefully monitored against several potential factors; advanced age-associated ARDS was the most important (Figure 4). Unfortunately, there is a severe paucity of high-quality data on the ARDS mechanisms affecting the two extremes of life. For instance, while ARDS post-traumatic events were most common in middle-aged adults, patients four years or younger and 65 years or older experienced the highest burden of ARDS-related mortality, and children were disproportionately affected by the incidence [62]. For these reasons, and to adequately understand the potential confounding factors in the host–pathogen interplay, we asked several research questions and determined the influence of ARDS, coinfections, and patient demographics before and after the COVID-19 pandemic on patients’ outcomes (Table 1a–e). These included age and gender specificities in ARDS fatalities as well as SARS-CoV-2 selective susceptibilities in gender differences. For instance, we examined age association before and during COVID-19 in admitted patients. We found no significant age-related differences in COVID-19 patients and no potential gender-based resistance or susceptibility to SARS-CoV-2 infection (Table 1) contrary to the common belief that being a man could be a risk for the virus. Nevertheless, advanced age was a factor in SARS-CoV-2 susceptibility irrespective of gender [63,64]. However, recent advances support host-specificity as a mechanism in the virus tropism, transmission dynamics, immune evasion, and virulence in different human population genetic structures [65]. Furthermore, as expected, it was unlikely from association studies (Table 1; *p* value 7.7) that ARDS pre-disposed one to bacterial co-infections in this study; however, the opposite seemed consistent, and bacteria are known to elicit immune reactions, causing cytokine storms that induce ARDS. More importantly, Kaplan–Meier survival analysis based on the hospital stay time (days) of patients with and without ARDS revealed that the former group stayed significantly longer (~60 days) (delayed mortality), supported by the high *p* value (*p* value = 0.0002). These findings are strong proof of concepts identified in this study that imply that increased hospital time was a risk for contraction of co-infection-induced SDRS, despite the delay in mortality due to supportive therapy. The first proof was the higher rates of COVID-19 and infectome-induced ARDS CFRs in co-infected patients, and the second was that the ~40-day period was the exact time when lethal intra-tracheal ventilations were used that exclusively increased the survival time of co-infected patients but did not help potentially non-infectious SDRS patients that stayed shorter time. For instance, ARDS was induced over time by prolonged infections of SARS-CoV-2 [66], bacterial infections [9,10,11,12,13,14,15,16,17,18,19,20,21], nosocomial CA-MRSA pneumonia, and sepsis with ARDS CFR from 56% to 63% [29,30,36,37]. These findings are critical in understanding potential pitfalls in patient care, as well as the diagnosis and treatment of ARDS and, specifically, the identification of its inducers. Thus, although delayed mortality can be achieved by supportive and empiric therapies, the specific identification of pathogens and chronic disorders is imperative in minimizing infectome-induced ARDS and ensuring the best patient treatment strategies for chronic disorders.

Despite enormous efforts, the mechanisms of coinfections in aggravating COVID-19 patient outcomes with and without underlying chronic disorders have not yet been clearly understood. In particular, accurate differential diagnosis between the cause of ARDSs, whether initiated by COVID-19, bacterial infections, and/or noninfectious ARDS, has not been well addressed. This is of paramount importance since it directs different specific treatment strategies for each cause. In this study, we addressed this issue in detail among the examined population, where 80% of ARDS patients with bacterial co-infections did not survive. These findings indicate the involvement of several subtle mechanisms during host–pathogen interactions. Therefore, from these results, we assumed that the lung injury was due to a potential cytokine storm provoked by a dynamic “infectome”. However, while cytokine storms were being reported [67] widely in the community-acquired *Staphylococcus strains* (CA-MRSA) during its pandemic a decade ago, their role in Gram-negatives has not yet been widely reported. To substantiate these results, stepwise investigations were necessary to confirm the influence of coinfections.

In independent investigations, we examined bacterial co-infections alone without underlying ARDS on the outcome of studied patients. Regarding overall survival analysis in these patients (*n* = 133), 31.3% died, while only 4.8% of patients without bacterial co-infection died. In other words, 95% of patients without co-infections survived the COVID-19 disease (Figure 3). This association of co-infection and COVID-19 fatality was highly associated with a higher death rate as indicated by the *p* value = 0.0121753241070998). We further determined the major bacterial pathogens that potentially predispose to ARDS and found that *Acinetobacter baumannii* and *Escherichia coli* (*E. coli*), either alone or in a mixed infection with *Klebsiella pneumoniae* (*K. pneumoniae*), were predominant species identified during ARDS attacks. The two pathogens, *A. baumannii* and *K. pneumoniae*, revealed aggressive profiles on patient characteristics, indicating a significant role in exacerbating the disease (Table 1). This is in agreement with our previous finding that only a few Gram-negative pathogens were identified that aggravated COVID-19 clinical profiles [67,68,69]. While selective SARS-CoV-2 coinfection by limited pathogens became increasingly evident, their role in the development of stroke and ARDS remained unclear. This has significant clinical implications in differential diagnosis and specific empiric therapy. Furthermore, SARS-CoV-2’s role in stroke has been widely proposed as evidence of viral tropism loci leading to ARDS; however, whether coinfections are involved is not fully understood. The nasal olfactory bulb expresses different transcript levels in nasal partitioning ration–inspiration (NRP1), ACE2, CD147, TMPRSS2, and Furin, accounting for smell and taste losses [70,71]. The higher expression levels of NRP1 in the SARS-CoV-20-infected cells of the olfactory epithelium imply a hematogenous spread—a potential route to stroke in COVID-19 patients. In addition, there are also well-established mechanisms in CA-MRSA superbug-induced cytokine storm production in necrotizing pneumonia. However, future vertical investigations for similar mechanisms in Gram-negative lung pathogenicity have become imperative since the growing outbreak of hypervirulent strains is rapidly circulating [72].

Another rather more important reason for differential diagnosis to understand the role of co-infection in COVID-19 is the potential molecular mimicry leading to co-protections against virus infections. While other viral co-infections are known to provide cross-protection against SARS-CoV-2 [73,74], this type of co-protection is rare in cases of bacterial co-infection, implying a risk of subtle bacterial virulence initiated by SARS-CoV-2. Some rare cases of cross-reactive epitomes with SARS-CoV-2 have been reported for proteomes of BCG, *Bordetella pertussis*, *Corynebacterium diphtheriae*, *Clostridium tetani*, *Hemophilus influenzae*, *Neisseria meningitidis*, and *Streptococcus pneumoniae* [75,76], and this implies that similar cases for the Gram-negatives identified in this study are possible. Thus, the proper diagnosis and management of ARDS–COVID-19 patients with underlying causes have become imperative since they are prone to co-infections by respiratory pathogens such as *Pseudomonas aeruginosa* as reported by Pezzuto et al.

## 5. Conclusions

In conclusion, for the first time to the best of our knowledge, we report on the frequencies of the associations of ARDS, coinfections, and patient demographics on COVID-19 patients’ outcomes. While ARDS and co-infections aggravated case fatality rates of COVID-19 patients, each either alone or in combination, advanced age was a factor in SARS-CoV-2 susceptibility irrespective of gender. More importantly, the “infectome” of *A. baumannii*, *E. coli*, and *Klebsiella pneumoniae* was identified in most ARDS cases and potentially might have provoked the attacks. Although delayed mortality was achieved through standard care and empiric therapies, the specific identification of pathogens and chronic disorders is imperative in minimizing infectome-induced ARDS and ensuring the best patient treatment strategies for chronic disorders. These findings have significant clinical implications and require a specific differential diagnosis of ARDSs induced by COVID-19 and bacterial infection. Future vertical investigation for similar mechanisms of cytokine-induced ARDS by Gram-negative pathogens is warranted due to the growing outbreak of hypervirulent strains, which are rapidly circulating [76] n the region. This study has limitations in that it is a single-center study confined to Ha’il hospitals; a large-scale investigation conducted in major national hospitals would gain more insights.

## Figures and Tables

**Table 1:** (**a**–**j**) Comparative analysis of multi-factors involved in patients’ outcomes, including ARDS, coinfections, and patient demographics before and after the COVID-19 pandemic in Ha’il, Saudi Arabia.

Characteristics				*p*-Value
***a.*** *Age profiles of patients before and after COVID-19 pandemic*				
	Mean	Std. Deviation	Std. Error Mean	
During COVID-19 *(total n = 149)*	57.154	15.5058	1.2703	
Before COVID-19 *(total n = 149)*	58.463	15.3286	1.2558	
***b.*** *Admitted patients’ gender profiles % (n)*				
	Female	Male		*p*-Value
During COVID-19	50% (*n* = 68)	50% (*n* = 81)		0.546
Before COVID-19	50% (*n* = 68)	50% (*n* = 81)		
Total	136	162		
***c.*** *Analysis of the association between admitted patients’ gender and acute respiratory distress syndrome % (n)*				
	Female	Male	Total	*p*-Value
No ARDS	91.9% (*n* = 125)	89.5% (*n* = 145)	90.6% (*n* = 270)	0.307
ARDS	8.1% (*n* = 11)	10.5% (*n* = 17)	9.4% (*n* = 28)	
Total	136	162	298	
***d.*** *Association of patients’ gender and COVID-19 fatality % (n)*				
	Female	Male	Total	*p*-Value
Death	20.6% (*n* = 28)	19.1% (*n* = 31)	19.8% (*n* = 59)	0.433
Survival	79.4% (*n* = 108)	80.9% (*n* = 131)	80.2% (*n* = 239)	
Total	136	162	298	
***e.*** *Whether acute respiratory distress syndrome predisposes to coinfections % (n)*				
	No Bacterial co-infection	Bacterial co-infection		*p*-Value
No ARDS	93.8% (*n* = 120)	88.2% (*n* = 150)	90.6% (*n* = 270)	0.77
ARDS	6.3% (*n* = 8)	11.8% (*n* = 20)	9.4 (*n* = 28)	
Total	128	170	298	
***f.*** *Survival analysis of ARDS conditions in COVID-19 patients % (n)*				
	Death	Survival	Total	
No ARDS	27.1% (*n* = 36)	72.9% (97)	100% (133)	
ARDS	75.0% (12)	25.0% (4)	100% (16)	
Total	32.2% (48)	67.8% (101)	100 (149)	
***g.*** Oxygen support and LALC recorded in SRDS- COVID-19 subjects with bacterial co-infections				
	*K. pneuomoniae*	*A. acinetobacer*	*E. coli*	
Intubations recorded	All were intubated	All were intubated	All were ventilated	
Liters oxygen (>4 L)	Variable but much more than 4	Always much more than 4	More than 4	
Ventilations recorded	Ventilated before intubation	Ventilated before intubation	Only ventilated	
***h.*** Time breathing assistance required	immediate	Mostly immediate	At later stages	
***i.*** LALC (low absolute Lymphocyte count	<5 (always <3–4)	<5 (always >3–4)	<5	
***j.*** Overall infiltration CXR (ground glass)	Yes	Yes	Not conclusive	

## Data Availability

There are no additional data deposited on any other site other than in this manuscript.
